# Health-related quality of life in patients with inborn errors of immunity: a bibliometric analysis

**DOI:** 10.3389/fimmu.2024.1371124

**Published:** 2024-03-07

**Authors:** Ningkun Xiao, Xinlin Huang, Wanli Zang, Sergey Kiselev, Mikhail A. Bolkov, Irina A. Tuzankina, Valery A. Chereshnev

**Affiliations:** ^1^Department of Immunochemistry, Institution of Chemical Engineering, Ural Federal University, Yekaterinburg, Russia; ^2^Laboratory for Brain and Neurocognitive Development, Department of Psychology, Institution of Humanities, Ural Federal University, Yekaterinburg, Russia; ^3^Postgraduate School, University of Harbin Sport, Harbin, China; ^4^Institute of Immunology and Physiology of the Ural Branch of the Russian Academy of Sciences, Yekaterinburg, Russia

**Keywords:** HRQOL, health-related quality of life, IEI, inborn errors of immunity, primary immunodeficiencies, quality of life, mental health, bibliometric analysis

## Abstract

**Background:**

Inborn Errors of Immunity (IEI) are characterized by a heightened susceptibility to infections, allergies, and various other health complications. Health-Related Quality of Life (HRQOL) in patients with IEI is a critical area of research that demands attention due to the impact of IEI on patients’ lives. This study utilized bibliometric methods, aiming to comprehensively explore the research content and hotspots in the field of HRQOL in patients with IEI.

**Methods:**

This bibliometric analysis utilized data from the Science Citation Index Expanded (SCIE) and Social Sciences Citation Index (SSCI) within the Web of Science core datasets up to January 1, 2024. The study focused on literature that addressed HRQOL in IEI patients, involving a total of 1,807 authors and 309 articles published across 112 journals. The analysis included publication volume and growth trends, country and institutional contributions, authorship, and journal analysis.

**Results:**

The research found that despite the importance of HRQOL in IEI, the volume of publications in this field remains consistently low, with no significant increase in trend. The USA leads in publication and citation volumes, reflecting a geographical imbalance in research contributions. Key journals in this field include the Journal of Clinical Immunology, Frontiers in Immunology, and the Journal of Allergy and Clinical Immunology. The study highlights that while treatments like hematopoietic stem cell transplants and gene therapy have improved patient IEI survival rates, they still often come with significant side effects impacting HRQOL. The analysis underlines the need for comprehensive HRQOL assessments in IEI, considering the physical and psychological impacts of treatments.

**Conclusions:**

This study represents a bibliometric analysis focusing on HRQOL in patients with. It underscores the need for more extensive and systematic research in this area, emphasizing the importance of a multidisciplinary approach. Despite advancements in medical treatments for IEI, there is a crucial need to focus on HRQOL to enhance patient satisfaction and overall well-being. The findings advocate for more personalized treatment plans and a better understanding of the psychosocial needs of patients with IEI to improve their quality of life.

## 1 Introduction

Inborn Errors of Immunity (IEI), formerly known as Primary Immunodeficiency (PID), represent a rare genetic disorder characterized by an individual’s increased susceptibility to infections, allergies, and a heightened risk of autoimmune diseases, inflammatory disorders, malignancies, and neurological complications, due to deleterious germline mutations (1–3). IEIs pose not only a severe threat to the patient’s life but also significantly reduce their quality of life (QoL). According to the 2022 update report by the Expert Committee of the International Union of Immunological Societies (IUIS) and recent studies, more than 500 different types of IEI have been described and identified (3–5). The primary treatment options for patients with IEI include Immune Globulin Replacement Therapy (IGRT) (6), Hematopoietic Stem Cell Transplant (HSCT) (7), Gene Therapy(GT) (8), Small Molecule Inhibitors (SMI) (9), antibiotics, antifungals (10) and thymus transplantation (11). The rapid advancement in high-throughput sequencing technologies and bioinformatics has greatly enhanced the identification and confirmation of new potential IEI-causing genes and phenotypes, enriching researchers’ and clinicians’ understanding of IEI and propelling advances in targeted gene-specific therapies and other precision medicine approaches (5, 12–15).

However, despite the evident improvement in the overall survival rate of the IEI population due to these treatment modalities and interventions, most patients with IEI still face challenges such as recurrent infections and frequent hospitalizations attributed to the chronic characteristics of IEI, treatment side effects, and the complexity of care management. This limits their normal physical and social activities (16, 17), making them more prone to anxiety (18), fatigue (19), depression (20), and increases their risk of cardiovascular diseases (CVD), malignancies, sleep disorders, and psychiatric conditions (21–23), severely impacting their physical and psychological health, and reducing their QoL and well-being. Therefore, it is imperative to extend the current clinical survival rate/disease parameter assessments to evaluate the overall health status of patients with IEI, aiding researchers, clinicians, and caregivers in better understanding the patient’s health condition.

Over the past few decades, the concept of Health-Related Quality of Life (HRQOL) as an effective measure of QoL has been refined and widely used in various studies related to human health (24). The measurement of HRQOL is dynamic and multidimensional, encompassing physical, behavioral, psychological, emotional, and social functioning domains that are of interest to researchers, healthcare professionals, policymakers, patients, and their families (24–26). Increasingly, studies demonstrate that HRQOL is a crucial indicator for assessing the effectiveness of interventions and the overall health of patients with chronic diseases. It goes beyond the direct measurement of general clinical indicators such as survival rates, adverse event rates, and mortality rates, focusing instead on a comprehensive understanding of the overall impact of disease and its interventions on a patient’s health life from the patient’s perspective (27, 28). Some research indicate that HRQOL measurements tailored to specific diseases or disorders can more effectively assess the positive or negative impacts of interventions on patients’ lives compared to general clinical opinions (29). This targeted approach explores factors affecting patients’ health lives, providing clear guidance for patients, doctors, and caregivers about treatment choices, management strategies, and targeted prevention related to treatment effectiveness. This reflects the ultimate goal of healthcare, which is not only to prolong life but also to improve the QoL for patients.

However, despite progress in research, there is still a lack of comprehensive analysis of authors, institutions, countries, and trends in the field of HRQOL research specifically for patients with IEI. Bibliometrics, a common method using mathematical and statistical techniques to explore and analyze literature, can analyze the associations between authors and institutions, revealing global research progress and trends (30). This helps researchers quickly identify current hotspots in the field, integrate research information, and promote communication and collaboration among researchers. Therefore, we will use bibliometric methods to integrate current HRQOL research in the field of IEI. We hope that research into the health life of patients with IEI will garner more attention from researchers, doctors, and caregivers, and enhance our understanding of the patients’ health status. This will enable the provision of tailored, targeted interventions and comprehensive care models, including socio-psychological support, counseling, and education programs, to holistically improve the HRQOL of patients with IEI.

## 2 Methodology

### 2.1 Literature retrieval and data collection

For our literature selection and collection, we utilized the core collection in Web of Science: Science Citation Index Expanded (SCIE) and Social Sciences Citation Index (SSCI). The search theme was divided into two parts, IEI and HRQOL: [TS=(“Primary Immunodeficiency Diseases”) OR TS=(“Primary Immunodeficiency”) OR TS=(“Primary Immunodeficiency Disorder”) OR TS=(“Inborn errors of immunity”)] AND [TS=(“Mental Health”) OR TS=(“Mental Hygiene”) OR TS=(“Psychological Health”) OR TS=(“Psychological Well-being”) OR TS=(“Psychological Well Being”) OR TS=(“Quality of Life”) OR TS=(“Life Quality”) OR TS=(“HRQOL”) OR TS=(“Health-Related Quality Of Life”) OR TS=(“Health Related Quality Of Life”)], with the search ending on January 1, 2024. The article types were limited to Articles and Reviews, and the language was set to English. Key information such as publication year, title, authors, abstracts, keywords, and journal names were exported in plain text format.

### 2.2 Data analysis

We used CiteSpace V5.7.R1 (Drexel University, Chaomei Chen, USA) and Excel 2019 (Microsoft, Washington, USA) for data deduplication, integration, and visual analysis. CiteSpace, a JAVA-based bibliometric program developed by Professor Chaomei Chen, facilitates visual analysis of researchers, institutions, trends, and hotspots (31). We also utilized website (https://bibliometric.com/) to analyze the collaboration relationships between different countries and regions. In the presented visual graphs, each node represents a different parameter, including countries, institutions, authors, and keywords. The weight of a parameter determines the size of its node, the heavier the weight, the larger the node. Nodes and lines are assigned different colors based on their clusters or timelines. The distance between any two nodes indicates their correlation, and the thickness of the connecting line represents the strength of the link.

## 3 Results

### 3.1 Publication volume and growth trends

**Figure 1:**
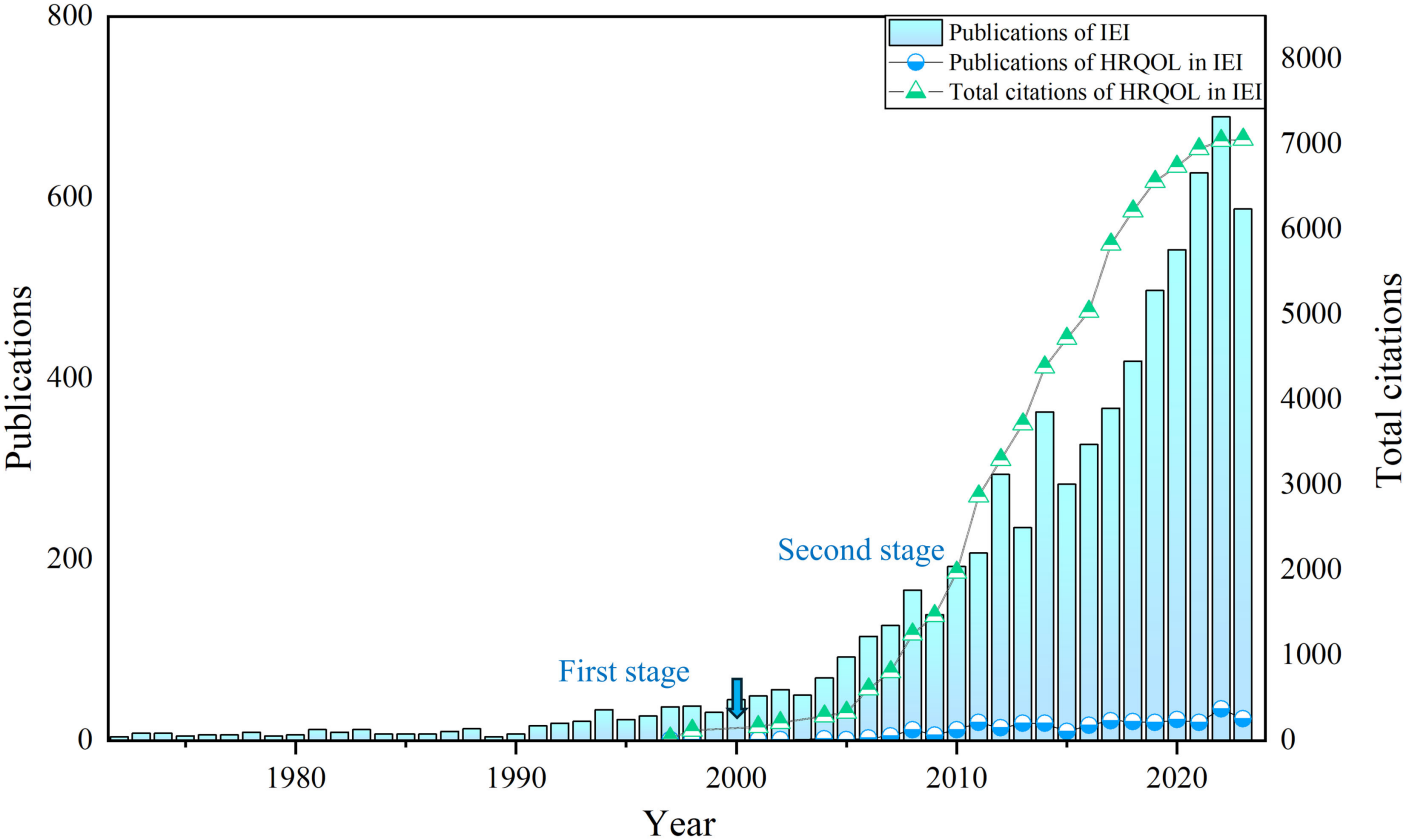
The number of annual publications and citations concerning HRQOL in IEI.

As of January 1, 2024, our search results revealed that 6,932 articles (original articles and reviews) related to IEI had been included in the Web of Science core datasets SCIE and SSCI. Out of these, 309 articles were related to HRQOL in IEI, accounting for only 4.46% of the publications in this field, indicating a relatively small proportion. As shown in **Figure 1**, since the first publication on HRQOL in patients with IEI in 1997, there has been no significant increase in the volume of publications in this field, with a consistent annual output of just over 20 articles in recent years. In terms of citation data, the field experienced slow growth in citations from 1972-2000, followed by a rapid increase after 2000. Overall, the current research on HRQOL in patients with IEI is limited in volume and lacks an explosive growth trend, indicating the need for more prospective research in this field.

### 3.2 Country and institutional analysis

**Figure 2:**
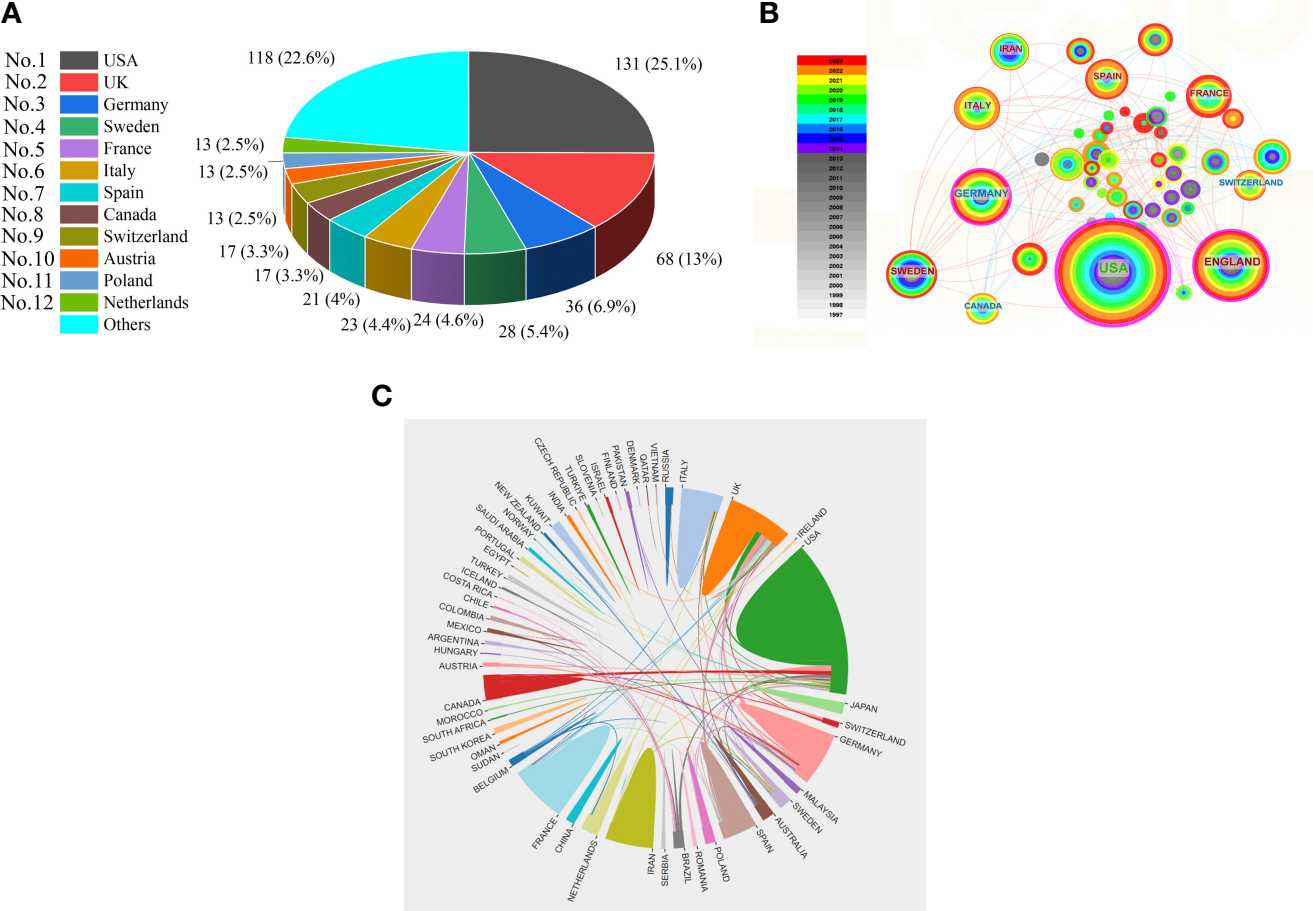
**(A)** The top 12 productive countries with publications concerning HRQOL in IEI; **(B)** The co-occurrence map of countries concerning HRQOL in IEI; **(C)** The international collaboration visualization map of countries/regions concerning HRQOL in IEI.

The research on HRQOL in IEI involved 52 countries/regions and 912 institutions. The USA led the field with 131 published articles, followed by the UK with 68 articles. Other countries with significant contributions include Germany (n = 36), Sweden (n = 28), France (n = 24), Italy (n = 23), Spain (n = 21), Canada (n = 17), Switzerland (n = 17), Austria (n = 13), Poland (n = 13), and the Netherlands (n = 13), with a collective contribution of 118 articles from other countries (see **Figure 2A** for details). It is noteworthy that most publications in this field originate from developed countries, indicating a significant gap in related research in developing countries and regions. In order to further explore the quantitative relationship between total GDP and GDP per capita and the number of publications, we further collected and organized the total GDP and GDP per capita of the top ten countries in terms of publication volume for the years from 2019 to 2022 and did the correlation analysis. Since the relationship between total GDP, GDP per capita, and the related number of publications does not conform to normal distribution and is not equally spaced fixed-distance data, we used the Kendall test, which showed that total GDP and the number of publications were strongly correlated (t = 0.674, sig. = 0.007 < 0.05), and that there was no correlation between GDP per capita and the number of publications (t = 0.135, sig. = 0.590 > 0.05) (see **Appendices 1**–**3** for details). **Figures 2B, C** display the collaborative relationships between countries and regions, with the top five countries in terms of centrality being the USA, UK, Germany, Sweden, and France. The USA holds a significantly leading position in the field with a centrality of 0.56. The USA, UK, Canada, Germany, and Japan have established close collaborative relationships.

**Figure 3:**
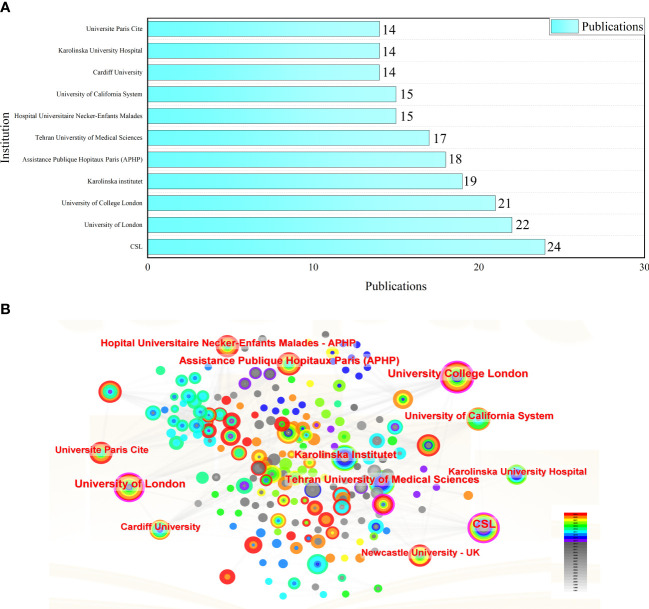
**(A)** The top 11 productive institutions with publications concerning HRQOL in IEI; **(B)** The analysis of research institutions concerning HRQOL in IEI.

The top ten institutions in terms of publication volume are the CSL Behring (pharmaceutical company) (n = 24), University of London (n = 22), University College London (n = 21), Karolinska Institute (n = 19), Assistance Publique Hopitaux Paris (n = 18), Tehran University of Medical Sciences (n = 17), Hospital Universitaire Necker-Enfants Malades (n = 15), University of California System (n = 15), Cardiff University (n = 14), and Karolinska University Hospital (n = 14) (see **Figure 3A** for details). The CSL Behring, University of London, and University College London, with centrality >0.1, have engaged in extensive and close collaborations with other institutions (see **Figure 3B** for details).

### 3.3 Author analysis

**Figure 4:**
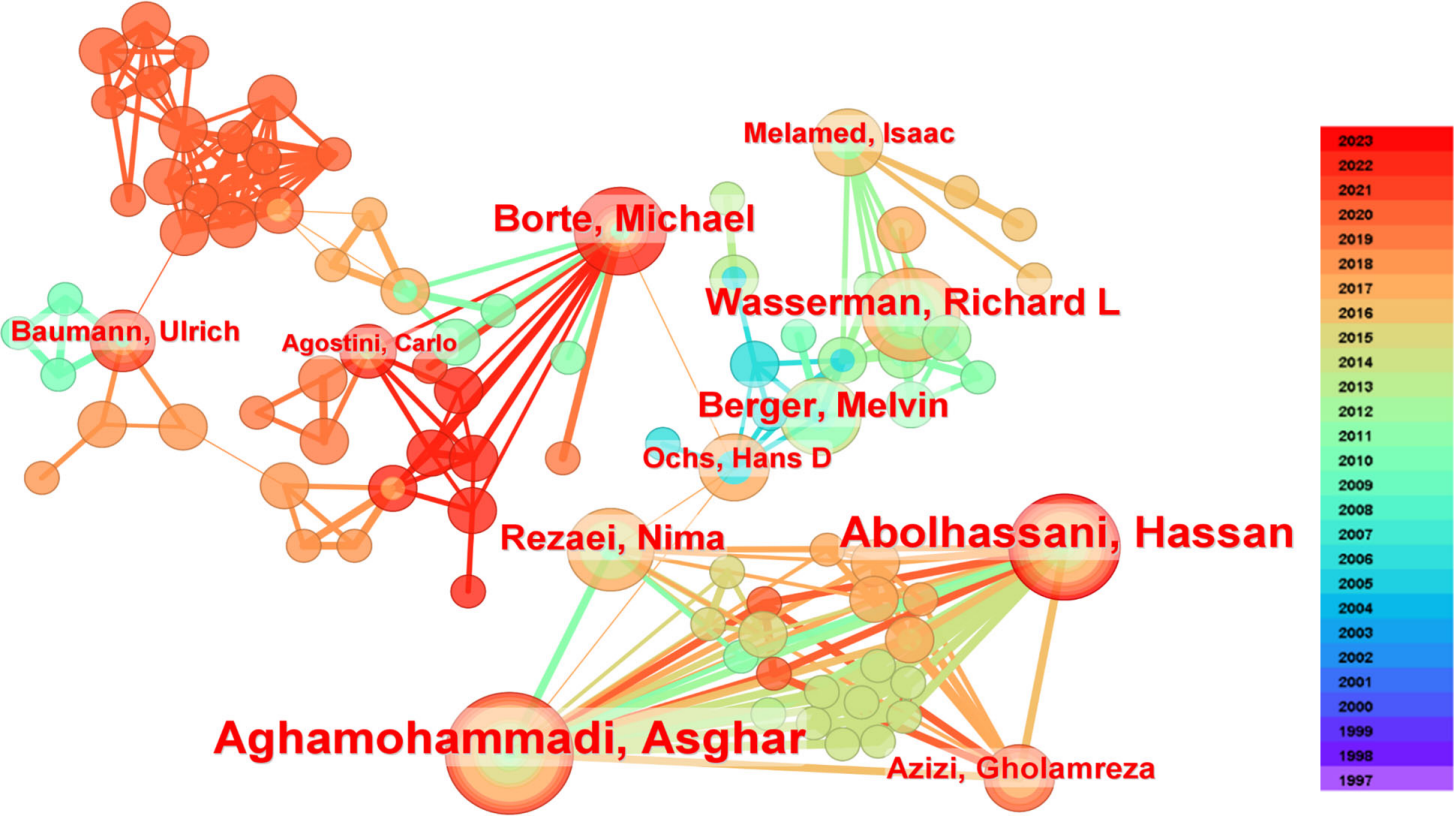
The co-occurrence authors’ map of HRQOL in IEI.

A total of 1,807 authors have contributed to the research on HRQOL in IEI. Among them, Asghar Aghamohammadi from Tehran University of Medical Sciences, Iran, leads with 15 published papers, followed closely by Hassan Abolhassani from Karolinska Institute, Sweden, with 13 publications. Michael Borte from Immune Defect Center Leipzig, Germany, Nizar Mahlaoui from Necker-Enfants Malades University Hospital, France, and Richard L Wasserman from the University of Texas Southwestern Medical School, USA, each have published 8 related papers, ranking third. Notably, among the top ten prolific authors, the USA has three authors, while Iran and Switzerland have two each, and France, Germany, and the UK each have one (see **Table 1** for details). **Figure 4** illustrates the collaborative relationships among authors. It shows a close collaboration between Asghar Aghamohammadi, Hassan Abolhassani, Nima Rezaei, and Gholamreza Azizi, as well as between Ochs HD, Berger Melvin, Richard L Wasserman, and Isaac Melamed, with Michael Borte and Carlo Agostini having a tighter collaboration.

**Table 1:** The top 10 productive and co-cited authors with publications concerning HRQOL in IEI.

Rank	Author	Affiliations and countries	Number of publications	Co-cited Author	Affiliations and countries	Citations
1	Asghar Aghamohammadi	Tehran University of Medical Sciences, Iran	15	Ann Gardulf	Huddinge University Hospital, Karolinska Institute, Sweden	131
2	Hassan Abolhassani	Karolinska Institutet, Sweden	13	Berger Melvin	Case Western Reserve University, USA	127
3	Michael Borte	Immune Defect Center Leipzig, German	8	Ochs HD	Seattle Children’s Hospital, USA	92
3	Nizar Mahlaoui	Necker-Enfants Malades University Hospital, France	8	Uwe Nicolay	Karolinska Institute, Sweden	75
3	Richard L Wasserman	University of Texas Southwestern Medical School, USA	8	Bonilla FA	Boston Children’s Hospital and Harvard Medical School, USA	75
6	Berger Melvin	Case Western Reserve University, USA	7	Jordan S Orange	Columbia University College, USA	75
6	Andrew R. Gennery	Great North Children’s Hospital in Newcastle, UK	7	Chapel HM	University of Oxford, UK	73
6	Jordan S Orange	Columbia University College, USA	7	S Jolles	University Hospital of Wales, UK	70
6	Nima Rezaei	Tehran University of Medical Sciences, Iran	7	Richard L Wasserman	University of Texas Southwestern Medical School, USA	65
10	Ann Gardulf	Huddinge University Hospital, Karolinska Institute, Sweden	6	Hassan Abolhassani	Karolinska Institute, Sweden	59

Although Asghar Aghamohammadi from Iran leads in publication output with 15 papers, Ann Gardulf from Huddinge University Hospital, Karolinska Institutet, Sweden, leads in citations with 131, followed by Berger Melvin from Case Western Reserve University, USA, with 127 citations, and Ochs HD from Seattle Children’s Hospital, USA, with 92 citations. Among the top ten most-cited authors, there are five from the USA, three from Sweden, and two from the UK, indicating these countries’ significant influence in the field (see **Table 1** for details).

### 3.4 Journal analysis

**Figure 5:**
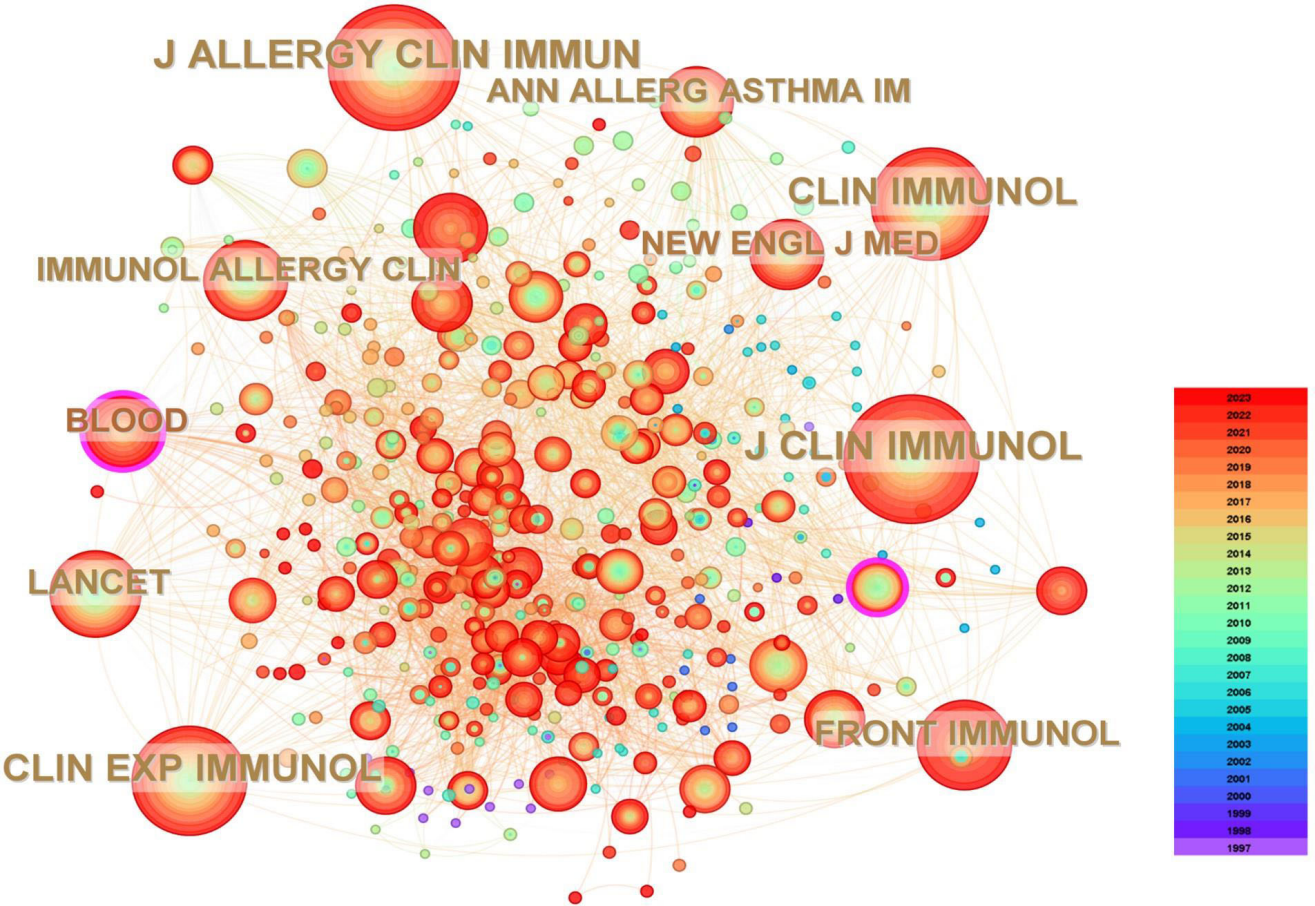
The co-cited journals’ map of HRQOL in IEI.

The 309 related articles were published in 112 journals. A visual analysis was conducted to showcase influential journals in the field. Among the publishing journals, the Journal of Clinical Immunology leads with the highest number of related publications (n = 67, 21.68%), followed by Frontiers in Immunology (n = 26, 8.41%), and the Journal of Allergy and Clinical Immunology (n = 20, 6.47%). The top ten journals collectively published 182 related articles, accounting for 58.90% of the total number of articles, indicating a significant concentration effect. In terms of citations, the Journal of Clinical Immunology leads with 282 citations, followed by the Journal of Allergy and Clinical Immunology with 266 citations, and Clinical Immunology with 214 citations. Looking at the top ten most-cited journals, eight are ranked in the Q1 quartile and two in the Q2 quartile according to the Journal Citation Reports (JCR), demonstrating their significant impact in the field (see **Table 2**, **Figure 5** for details).

**Table 2:** The top 10 most productive journals and co-cited journals concerning HRQOL in IEI.

Rank	Journals	Number of publications	Number of citations	Average number of citations	IF (2023), JCR quantile	Co-cited journals	Number of co-citations	Centrality	IF (2023), JCR quantile
1	Journal of Clinical Immunology	67	463	6.91	9.1, Q1	Journal of Clinical Immunology	282	0.01	9.1, Q1
2	Frontiers in Immunology	26	27	1.04	7.3, Q1	Journal of Allergy and Clinical Immunology	266	0.01	14.2, Q1
3	Journal of Allergy and Clinical Immunology	20	74	3.7	14.2, Q1	Clinical Immunology	214	0.01	8.6, Q1
4	Clinical and Experimental Immunology	18	204	11.33	4.6, Q2	Clinical and Experimental Immunology	208	0.01	4.6, Q2
5	Immunotherapy	12	21	1.75	2.8, Q4	Lancet	140	0.07	168.9, Q1
6	Expert Review of Clinical Immunology	9	17	1.89	4.4, Q2	Frontiers in Immunology	123	0.01	7.3, Q1
7	Clinical Immunology	8	107	13.38	8.6, Q1	Annals of Allergy Asthma & Immunology	113	0.05	5.9, Q2
8	Annals of Allergy Asthma & Immunology	8	88	11	5.9, Q2	Blood	107	0.12	20.3, Q1
9	Allergy Asthma and Clinical Immunology	7	32	4.57	2.7, Q3	New England journal of medicine	105	0.06	158.5, Q1
10	Allergologia Et Immunopathologia	7	13	1.86	1.8, Q4	Pediatrics	98	0.03	8, Q1

### 3.5 Reference analysis

**Figure 6:**
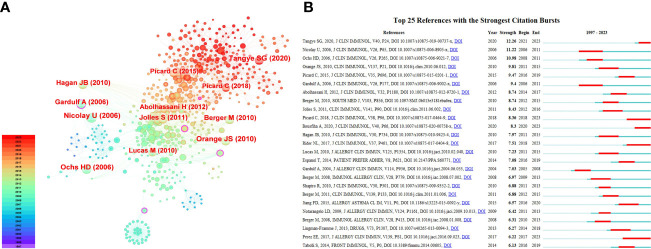
**(A)** The analysis of references related to HRQOL in IEI; **(B)** The top 25 references with strong citation bursts.

**Table 3** lists the top ten most-cited references related to HRQOL in IEI. The most cited is the 2019 update by the International Union of Immunological Societies Expert Committee (IUIS) on IEI, emphasizing the goals of raising awareness, identifying IEI, promoting optimal treatment methods, and supporting research in the field of immunological diseases (2). Following this is the 2015 update by the IUIS (32), and an article by Ochs HD et al. (33) systematically introducing Wiskott-Aldrich syndrome, highlighting the major challenges and significance of managing this condition. Six out of the top ten most-cited articles were published in the Journal of Clinical Immunology, with two each in the Journal of Allergy and Clinical Immunology and Clinical Immunology, underlining the importance of these three journals in the field. **Figure 6A** displays the top ten cited references in the Web of Science core collection, with different colors representing the publication time of the articles - the closer to red, the more recent the publication, and the closer to purple, the older. **Figure 6B** shows the 25 articles with the highest citation burst intensity. The earliest burst started in 2006, with the highest burst intensity being 12.26 for Tangye SG et al.’s 2020 article in the Journal of Clinical Immunology on “Human Inborn Errors of Immunity: 2019 Update from the International Union of Immunological Societies Expert Committee” (2). The years 2006, 2010, and 2020 witnessed more frequent high-citation bursts, indicating a heightened focus on HRQOL research in IEI during these years.

**Table 3:** The top 10 cited references concerning HRQOL in IEI.

Rank	Title	First Author	Year	Journal	Citations in Google scholar
1	Human Inborn Errors of Immunity: 2019 Update on the Classification from the International Union of Immunological Societies Expert Committee	Tangye SG	2020	Journal of Clinical Immunology	1127
2	Primary Immunodeficiency Diseases: An Update on the Classification from the International Union of Immunological Societies Expert Committee for Primary Immunodeficiency 2015	Picard C	2015	Journal of Clinical Immunology	836
3	The Wiskott-Aldrich syndrome	Ochs HD	2006	Journal of Allergy and Clinical Immunology	496
4	Infection outcomes in patients with common variable immunodeficiency disorders: Relationship to immunoglobulin therapy over 22 years	Lucas M	2010	Journal of Allergy and Clinical Immunology	469
5	Impact of trough IgG on pneumonia incidence in primary immunodeficiency: A meta-analysis of clinical studies	Orange JS	2010	Clinical Immunology	452
6	Health-Related Quality of Life and Treatment Satisfaction in North American Patients with Primary Immunedeficiency Diseases Receiving Subcutaneous IgG Self-Infusions at Home	Nicolay U	2006	Journal of Clinical Immunology	221
7	Rapid Subcutaneous IgG Replacement Therapy is Effective and Safe in Children and Adults with Primary Immunodeficiencies—A Prospective, Multi-National Study	Gardulf A	2006	Journal of Clinical Immunology	219
8	Home-Based Subcutaneous Immunoglobulin Versus Hospital-Based Intravenous Immunoglobulin in Treatment of Primary Antibody Deficiencies: Systematic Review and Meta Analysis	Abolhassani H	2012	Journal of Clinical Immunology	198
9	Efficacy and Safety of a New 20% Immunoglobulin Preparation for Subcutaneous Administration, IgPro20, in Patients with Primary Immunodeficiency	Hagan JB	2010	Journal of Clinical Immunology	139
10	Efficacy and safety of Hizentra^®^ in patients with primary immunodeficiency after a dose-equivalent switch from intravenous or subcutaneous replacement therapy	Jolles S	2011	Clinical Immunology	125

### 3.6 Keywords and hotspot analysis

**Figure 7:**
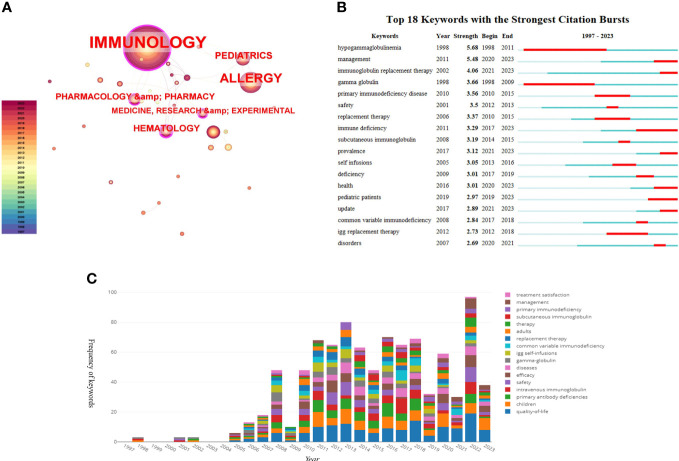
**(A)** The analysis of research fields; **(B)** The top 18 keywords with strong citation bursts; **(C)** The map of top 17 keywords over time.

The 309 articles were published across 36 fields related to HRQOL in IEI, with key areas being immunology, allergy, pediatrics, hematology, health care sciences, and health policy (see **Figure 7A**). This demonstrates that focusing on HRQOL in IEI is a multidisciplinary endeavor, requiring collaboration across various fields to promote its development.

Keyword analysis quickly identifies the research content and hotspots within the field. As shown in **Figures 7B, C**, the most common keyword is “quality of life,” appearing 177 times, indicating the focus and goal of the research, which is to investigate and improve the QoL of IEI patients. This is followed by “primary immunodeficiency (PID),” appearing 124 times, and “children,” appearing 98 times, indicating a greater focus on pediatric patients with IEI. “Intravenous immunoglobulin” appears 85 times, reflecting the long-standing treatment method for patients with IEI as well as the frequency of antibody deficiency diseases among IEI and the impairment of humoral immunity in combined immunodeficiency diseases. Other common keywords include “safety” and “efficacy,” showing the researchers’ focus on the treatment methods for patients with IEI. In the analysis of keyword burst intensity, terms like “hypogammaglobulinemia,” “management,” “immunoglobulin replacement therapy,” and “gamma globulin” are among the top in burst strength. Notably, keywords such as “hypogammaglobulinemia” and “gamma globulin” had bursts from 1998 to 2011, while “immunoglobulin replacement therapy,” “replacement therapy,” and “management” started bursting from 2015.

These trends reflect the evolving landscape of research in the field of IEI and HRQOL, highlighting the progress and shifts in focus over time, from the understanding and identification of IEI to the optimization of treatment and management strategies. The concentration on improving the QoL for patients with IEI, particularly in pediatric cases, underscores the importance of this research in addressing the comprehensive needs of this patient population. The attention given to treatment modalities like immunoglobulin replacement therapy and the emphasis on management strategies reveal an ongoing commitment to enhancing the effectiveness and safety of treatments for IEI. These insights, derived from the analysis of top-cited articles and keyword trends, are crucial for guiding future research and clinical practices in this vital area of healthcare.

## 4 Discussion

### 4.1 General information

As of January 1, 2024, 1,807 authors have published 309 articles on HRQOL in patients with IEI across 112 journals in the Web of Science core datasets SCIE and SSCI. Despite the first study on HRQOL in patients with IEI being published in 1997, the field has not seen a significant breakthrough in publication volume, maintaining a consistent trend of just over 20 publications annually. This indicates that, while the overall health status of patients with IEI is important, the current focus in the field of IEI remains on the determination of genetic and clinical phenotypes of patients with IEI, as well as their treatment and care, with relatively few researchers venturing into the study of HRQOL in patients with IEI.

From the available data, the USA leads in both publication and citation volumes in the field of HRQOL in patients with IEI and has extensive collaborations with countries worldwide. Among the top ten countries/regions in publication volume, nine are developed countries/regions, highlighting an imbalance in research across countries and indicating that socio-economic factors are one of the barriers to studying the HRQOL of patients with IEI. We also looked at the connection between total GDP, GDP per capita, and the number of publications. The results show that total GDP and the number of publications is strongly related (t = 0.674, sig. = 0.007 < 0.05), but GDP per capita and the number of publications is not related (t = 0.135, sig. = 0.590 > 0.05). This result needs to be interpreted with caution because we only analyzed the top ten countries in terms of the number of publications, which is not a large enough sample size and still needs to be further explored in the future. It is very important for us to know the potential dynamics of each economic indicator and their relationship with research output, which can help us understand the inputs, outputs, output efficiency, etc. of scientific research in the field in each country and region. The total GDP, on the other hand, is a measure of a country’s overall economic output, which can directly affect its ability to fund research (i.e., the amount of money invested in research). Countries with higher GDP tend to have a large number of high-quality universities, hospitals, and research institutions and are able to allocate more resources to support large research institutions, maintain advanced research infrastructure, and lead and promote international exchanges and collaborations between countries, which undoubtedly promotes the publication of papers in the field. Although the results show no correlation between GDP per capita and the number of publications in the field, it is clear that countries with higher GDP per capita are more capable of focusing on HRQOL and that developing countries and regions still need to strengthen their cooperation and exchange with these countries in order to improve the HRQOL of patients with IEI. Institutions like the CSL Behring, University of London, University College London, and Karolinska Institute are leading in publication volume and citations, signifying their forefront position in HRQOL research in IEI.

From an author perspective, Asghar Aghamohammadi from Tehran University of Medical Sciences, Iran, has the highest number of publications, with 15 related articles. He has conducted extensive research on the HRQOL of patients with IEI in Iran, including studies on the economic costs of different treatment methods for patients with IEI and the impact of healthcare utilization costs on the HRQOL of patients with IEI and their families (34, 35). Ann Gardulf from Huddinge University Hospital, Karolinska Institute, Sweden, has the highest number of citations. Ann Gardulf’s research includes comparisons of IgG replacement therapy and home-based self-therapy on the HRQOL of patients with IEI, investigations into the treatment satisfaction of patients receiving immunoglobulin replacement therapy, and summaries of risk factors affecting the HRQOL of patients with IEI (36–38). These studies have greatly promoted the academic community’s attention to the HRQOL of patients with IEI and the more scientific management and care of these patients. Overall, there is close collaboration between high-output and highly cited authors, which has accelerated the rapid development of research in this field.

In the field of HRQOL for IEI, the Journal of Clinical Immunology leads the way with 61 published articles, making it the most prolific journal in this area. It is followed closely by Frontiers in Immunology (n = 26, 8.41%) and the Journal of Allergy and Clinical Immunology (n = 20, 6.47%). In terms of citations, the Journal of Clinical Immunology, Journal of Allergy and Clinical Immunology, and Clinical Immunology occupy the top three positions. These journals, being specialized in the IEI field, play a crucial role in disseminating and promoting research in this area. The analysis of journals also aids researchers in quickly identifying suitable publication venues, thus saving time in the journal selection process.

Co-citation analysis helps to gauge the level of interrelatedness and importance among articles. Six of the top ten most-cited articles were published in the Journal of Clinical Immunology, with the Journal of Allergy and Clinical Immunology and Clinical Immunology each publishing two. This further emphasizes the central position of these journals in the field. Among these ten articles, two are updates from the International Union of Immunological Societies Expert Committee on IEI pathogenic genes, phenotypes, and future research focuses (2, 32), our directly research the HRQOL, treatment safety, and satisfaction of IEI patients (39–42), and the remaining four are reviews or meta-analyses related to HRQOL in patients with IEI (33, 43–45).

Keywords are often summaries of the core ideas of an article and can reflect the hot research topics in a specific field. Analyzing the evolution of keywords allows for understanding the shifts in research focus and future trends in the field. In HRQOL research for IEI, prominent areas include immunology, allergy, pediatrics, hematology, health care sciences, and health policy, corresponding to the frequent infections and healthcare needs in patients with IEI. “Quality of life” is the most recurrent keyword, being the central focus of this research. Keywords like “safety” and “efficacy” indicate an emphasis on the effectiveness and safety of treatment methods for patients with IEI. The keyword bursts also reveal that the main treatment approaches for patients with IEI include replacement therapy and management strategies. These keywords underscore the evolving landscape in the treatment and management of IEI, highlighting the progress towards more comprehensive and effective care approaches.

### 4.2 In the aspect of HRQOL for patients with IEI

It is evident that while current treatments like HSCT, antibiotic therapy, and gene therapy have significantly improved patient survival rates, they inevitably come with side effects that severely impact QoL of patients. For instance, common treatments like Immunoglobulin Replacement Therapy (IRT) require regular infusions, which, while lifesaving, can lead to side effects such as headaches, fever, chills, and fatigue, potentially resulting in severe adverse reactions 46). HSCT, though offering potential curative outcomes, carries the risk of serious complications like Graft-versus-Host Disease (GVHD), infections, and organ damage (47). Additionally, the complexity and associated risks of identifying suitable donors for HSCT often led to treatment delays. Gene therapy, aimed at targeting specific genetic anomalies in IEI, is still in its nascent stage.

These treatments, while advancing patient care, have left many patients with IEI grappling with repeated infections and frequent hospitalizations due to their side effects and the complexity of their management. This significantly affects their physical and psychological health, reducing their overall QoL and well-being. Therefore, a systematic investigation of the health status of IEI patients is essential, which can help us better understand their health conditions.

From the 309 studies collected, it is evident that compared to healthy children and adults, patients with IEI generally have a significantly poorer overall health status. Additionally, even compared to other chronic disease patients, patients with IEI have a lower self-perception of health, which is particularly concerning given studies showing the influence of perceived health on relative mortality risks (16). Patients with IEI also face more severe mental health challenges than the healthy population, such as loneliness, anxiety, and depression, which severely affect their health status. Previous research has shown that psychosocial factors significantly impact the incidence, QoL, and mortality rates of chronic diseases (48, 49).

These issues highlight the importance of focusing on the HRQOL of patients with IEI. However, despite the growing emphasis on assessing HRQOL in fields like oncology for patients with leukemia and cancer, its application in IEI is not widespread. This is primarily due to the difficulty in conceptualizing and assessing HRQOL due to the complex interplay of factors that affect it, including physical health, mental health, family relationships, social relationships, and environment. Furthermore, specific disease assessments often require a combination of generic and disease-specific scales to effectively evaluate a patient’s current HRQOL. The development of specific assessment scales for some diseases is not yet fully advanced, posing significant challenges to the widespread application of HRQOL assessments.

There is a clear need for more comprehensive and nuanced approaches to assess and improve the HRQOL of patients with IEI. This includes developing and utilizing both generic and disease-specific HRQOL measures that accurately reflect the unique challenges faced by patients with IEI. Additionally, addressing the broader psychosocial factors impacting these patients is crucial. This means not only focusing on the physical aspects of the disease but also providing adequate psychological and social support to improve their mental health and overall well-being.

Moreover, the disparities in research and focus between developed and developing countries highlight the need for a global effort to improve understanding and management of IEI. This would involve collaborative research, sharing of best practices, and equitable access to treatment and care, ensuring that all patients with IEI, regardless of their geographical location, have the opportunity to achieve a better quality of life.

In conclusion, while significant strides have been made in treating IEI, there is a pressing need to place a greater emphasis on the HRQOL of patients. This involves not only advancing medical treatments but also developing robust mechanisms for HRQOL assessment and addressing the psychological and social needs of these patients. As the field continues to evolve, incorporating these dimensions into care and research will be critical for improving the overall health and well-being of patients with IEI.

## 5 Limitations

In this study, we relied solely on data from the SCIE and SSCI within the Web of Science core datasets, excluding non-English language articles. This inevitably led to some information being overlooked, such as articles included in databases like Scopus and PubMed that are not covered in the Web of Science core collections and relevant articles published in languages other than English. This limitation potentially weakens the perceived impact of HRQOL research in the IEI field. It is known that journals in languages like Chinese, Russian, and Spanish also contain significant research on HRQOL in patients with IEI. However, we believe that using the Web of Science, a globally recognized authoritative database, as our data source does not compromise the validity of our conclusions. Additionally, our analysis of article impact focused on citation counts, which may underrepresent the influence of more recently published articles.

## 6 Conclusion

To our knowledge, this is the first bibliometric study specifically addressing HRQOL in patients with IEI. We included articles from the SCIE and SSCI databases of the Web of Science, involving 1,807 authors and 112 journals, resulting in 309 articles on HRQOL in patients with IEI. Overall, the volume of publications in this field remains low, with no apparent trend towards a surge in research.

Nonetheless, we firmly believe that focusing on the HRQOL of patients with IEI is critically important and necessary. Understanding the physical and psychological impacts of disease treatment on patients with IEI and comparing the effects of different treatment modalities on the HRQOL of patients and their family members can lead to targeted improvements in patient satisfaction. Early detection of psychological issues in patients with IEI can also facilitate better social interaction, reduce feelings of isolation, and overall enhance their satisfaction with life. We are confident that systematic research into the HRQOL of patients with IEI can lead to more personalized treatment plans, increase patient compliance, and improve their overall sense of well-being.

## Data availability statement

The original contributions presented in the study are included in the article/supplementary material. Further inquiries can be directed to the corresponding authors.

## Author contributions

NX: Writing – review & editing, Writing – original draft, Visualization, Validation, Software, Resources, Project administration, Methodology, Investigation, Formal analysis, Data curation, Conceptualization. XH: Writing – review & editing, Visualization, Validation, Formal analysis, Data curation. WZ: Writing – review & editing, Methodology, Formal analysis, Data curation. SK: Writing – review & editing, Validation, Supervision. MB: Writing – review & editing, Validation, Supervision. IT: Writing – review & editing, Validation, Supervision. VC: Writing – review & editing, Validation, Supervision.

## Appendix

**Appendix 1:** Total GDP and GDP per capita of the top 10 countries in the publication.

Year	USA	UK	Germany	Sweden	France	Italy	Spain	Canada	Switzerland	Austria
2022	25.44	3.082	4.072	0.591	2.783	2.01	1.398	2.14	0.808	0.471
2021	23.32	3.123	4.26	0.64	2.958	2.114	1.427	2.001	0.801	0.48
2020	21.06	2.706	3.89	0.547	2.639	1.897	1.277	1.648	0.74	0.435
2019	21.38	2.858	3.888	0.534	2.729	2.011	1.394	1.744	0.721	0.445
Average GDP/trillion dollars	22.800	2.942	4.028	0.578	2.777	2.008	1.374	1.883	0.768	0.458
2022	76343	45461	48756	55873	42350	34084	29800	54966	88760	47585
2021	70219	46422	51204	61143	43659	35770	30104	52359	87460	45409
2020	63529	40347	46773	52838	39055	31919	26960	43350	84460	43500
2019	65120	42747	46794	51939	40495	33674	29582	46347	87121	46647
GDP per capita/dollars	68802.75	43744.25	48381.75	55448.25	41389.75	33861.75	29111.5	49255.5	86950.25	45785.25
Publications	131	68	36	28	24	23	21	17	17	13

Data from internet. There may be some bias from different data sources.

**Appendix 2:** Correlation analysis between total GDP and number of publications.

			Average GDP	Publications
Kendall tau_b	Average GDP	Correlation coefficient	1.000	.674^**^
		Sig. (two-tailed)	.	.007
		N	10	10
	Publications	Correlation coefficient	.674^**^	1.000
		Sig. (two-tailed)	.007	.
		N	10	10

**At the 0.01 level (two-tailed), the correlation was significant.

**Appendix 2:** Correlation analysis between GDP per capita and number of publications.

			Publications	GDP per capita
Kendall tau_b	Publications	Correlation coefficient	1.000	.135
		Sig. (two-tailed)	.	.590
		N	10	10
	GDP per capita	Correlation coefficient	.135	1.000
		Sig. (two-tailed)	.590	.
		N	10	10
